# Wellbeing support for foundation doctors during COVID-19 in GHNHSFT

**DOI:** 10.1192/bjo.2021.510

**Published:** 2021-06-18

**Authors:** Abbi Graham, Anna-Marie Dale

**Affiliations:** Gloucestershire Hospitals NHS Foundation Trust

## Abstract

**Aims:**

The COVID-19 pandemic highlighted the importance of wellbeing amongst healthcare professionals. Medical professionals, notably junior doctors, are at increased risk of developing poor mental health and burnout. The GMC Barometer Study in 2020 showed that 32% of doctors found the first wave of the COVID-19 pandemic detrimental to their wellbeing and mental health.

The aim of this quality improvement project was to assess and improve hospital wellbeing support available to foundation doctors within Gloucestershire Hospitals NHS Foundation Trust (GHNHSFT) by learning and reflecting on the impact of COVID-19.

**Method:**

After identifying a lack of resources within GHNHSFT, wellbeing information boards were displayed in communal areas and distributed by email. These encompassed trust wide support, practical information including childcare and relaxation resources concentrating on mindfulness, exercise and culture. A survey of foundation doctors was completed to assess doctors’ focus and approach to wellbeing. Questions assessed influential factors in maintaining wellbeing, access to current hospital resources and future interventions.

**Result:**

94% of respondents recognised that their focus on wellbeing increased during COVID-19. One third of foundation doctors found it challenging to maintain their wellbeing, with 40% reporting difficulty accessing hospital support and advice. The most important factors foundation doctors identified in maintaining wellbeing were exercise, cooking and baking, and social networks. Colleagues were a significant source of wellbeing support, followed by notice boards, email resources and social media.

**Conclusion:**

COVID-19 highlighted the importance and burden on wellbeing of foundation doctors, with a significant number struggling to access support. Future recommendations include the use of a ‘buddy system’, regular and accessible exercise classes and improved communication of wellbeing support and resources to staff members.

Buddy systems have already shown success amongst teams however it is important these are accessible to all foundation doctors and universally offered within the trust. A weekly yoga class is being reintroduced to be available to all doctors.

A particular focus has been the development of a health and wellbeing section to feature in the trusts weekly communications, with the aim to regularly signpost staff to ongoing wellbeing resources and support.

Social networking and media were highlighted as important in both maintaining wellbeing and accessing resources. A future goal is to develop an official GHNHSFT Instagram or Twitter account focused on wellbeing. We hope to continue to learn from the impact of COVID-19, improving the availability of wellbeing support at GHNHSFT that will continue into the future.

